# Decreased expression of METTL14 predicts poor prognosis and construction of a prognostic signature for clear cell renal cell carcinoma

**DOI:** 10.1186/s12935-020-01738-2

**Published:** 2021-01-11

**Authors:** Yi Wang, Rong Cong, Shouyong Liu, Bingye Zhu, Xing Wang, Qianwei Xing

**Affiliations:** 1 Department of Urology, Affiliated Hospital of Nantong University, No. 20 West Temple Road, Nantong, 226001 Jiangsu Province China; 2 Department of Urology, The First Affiliated Hospital of Nanjing Medical University, Nanjing, 210029 Jiangsu Province China; 3 Department of Urology, Zhenjiang Hospital of Chinese Traditional and Western Medicine, Zhenjiang, 212000 Jiangsu Province China

**Keywords:** METTL14, N6-Methyladenosine, Clear cell renal cell carcinoma, Overall survival, Risk score

## Abstract

**Background:**

METTL14, as one of N6-methyladenosine (m6A) related genes, has been found to be associated with promoting tumorigenesis in different types of cancers. This study was aimed to investigate the prognostic value of METTL14 in clear cell renal cell carcinoma (ccRCC).

**Methods:**

We collected ccRCC patients’ clinicopathological parameters information and 13 m6A related genes expression from The Cancer Genome Atlas (TCGA) database. Univariate and multivariate Cox regression analyses were conducted to investigate whether METTL14 could serve as an independent factor correlated with overall survival (OS). Gene Set Enrichment Analysis (GSEA) was carried out to identify METTL14-related signaling pathways. Moreover, a risk score (RS) was calculated to predict the prognosis of ccRCC. Quantitative real-time PCR (qRT-PCR) was also utilized to verify the expression of METTL14 in clinical specimens.

**Results:**

Differently expressed m6A related genes were identified between ccRCC tissues and normal tissues. Therein, METTL14 was lowly expressed in ccRCC tissues and verified by qRT-PCR (all p < 0.01). Survival analysis indicated that high expression of METTL14 was associated with better OS (p = 1e−05). GSEA results revealed that high METTL14 expression was enriched in ERBB pathway, MAPK pathway, mTOR pathway, TGF-β pathway and Wnt pathway. Moreover, METTL14 was proved to be an independent prognostic factor by means of univariate and multivariate Cox regression analyses. Nomogram integrating both the METTL14 expression and clinicopathologic variables was also established to provide clinicians with a quantitative approach for predicting survival probabilities of ccRCC. Furthermore, a METTL14-based riskscore (RS) was developed with significant OS (p = 6.661e−16) and increased AUC of 0.856. Besides, significant correlated genes with METTL14 were also provided.

**Conclusions:**

Our results indicated that METTL14 could serve as a favorable prognostic factor for ccRCC. Moreover, this study also provided a prognostic signature to predict prognosis of ccRCC and identified METTL14-related signaling pathways.

## Background

Kidney cancer ranks the 6th and 8th most common cancer in males and females respectively, with approximately 73,750 estimated new cases and 14,830 newly estimated mortality in the United States, 2020 [1]. Clear cell renal cell carcinoma (ccRCC), as the most common histological type of RCC, accounts for approximately 85% of all primary renal neoplasms [2]. The overall 5-year survival rate of RCC is 74% and 16% of patients have been diagnosed with local invasion or distant metastasis, resulting in a poorer prognosis [3]. Hence, the early diagnosis and treatment of ccRCC are imperative and challenging. There is an urgent need to clarify the possible mechanisms underlying the development of ccRCC, to discover novel biomarkers for early screening, and to develop therapeutic targets.

Methylation of the N6 position of adenosine (m6A) has been found to be widespread throughout nuclear mRNA, rRNA, tRNA, and some snRNA of eukaryotes [4, 5]. The modified RNAs, especially mRNA, play critical roles in the post-transcriptional regulation of gene expression. As the most common type of mRNA modification in eukaryote, m6A was proved to be highly conserved, as well as in human and mouse. The m6A sites were found mostly located in 3’ untranslated regions (UTR) or near the stop codon [6, 7]. RNA m6A modification has been shown to play vital for RNA splicing, export, stability, regulation of RNA-protein interaction and gene expression [4, 8–10]. m6A is a reversible modification of mRNA mediated by the dynamic activities of m6A “writers” and “erasers” [11]. The genes of “writers” compose a mRNA methyltransferase enzyme complex, including METTLE3, METTL14, WTAP, KIAA1429, ZC3H13 and RBM15, while the reversible process is catalysed by m6A “erasers”, FTO and ALKBH5, conducting demethylation of m6A. In order to exert functions of m6A modifications, mRNA m6A sites can be recognized by RNA-binding proteins “readers”, including YTHDF1, YTHDF2, YTHDC1, YTHDC2 and HNRNPC.

Recently, m6A modification has been found to regulate embryonic stem cells and myeloid differentiation, spermatogenesis, obesity, sex determination, neuronal development [12–17]. The m6A regulatory genes have been shown to plays an oncogenic role in both breast cancer and acute myeloid leukemia by means of cancer stem cell maintenance and dedifferentiation of cancer cells [4, 18]. Previous study has suggested that METTL3 might be essential in lung cancer cells by directly promoting translation independently of methyltransferase activity and downstream m6A reader proteins [19]. According to another study, it has been proved that METTL14, a major RNA N6-adenosine methyltransferase, suppresses the metastatic potential of hepatocellular carcinoma by modulating m6A-dependent primary microRNA processing [20]. Therein, METTL14, as one of N6-methyladenosine (m6A) related genes, has been found to be associated with promoting tumorigenesis in different cancers. Recent studies reported that bioinformatics analysis could provide possible functions of many different novel genes in cancer research [21]. Hence, this study was aimed to investigate the prognostic value of METTL14 in ccRCC by bioinformatics analysis. Furthermore, we also provided a prognostic signature to predict prognosis of ccRCC patients and related signal pathways regulated by METTL14 by means of gene set enrichment analysis (GSEA).

## Materials and methods

### Data acquisition

All of these normalized RNA-seq data and clinical data of kidney renal clear cell carcinoma (KIRC) patients were obtained from TCGA website (http://cancergenome.nih.gov/) by TCGA-assembler. Finally, we identified the gene expression profiles and clinical data of 539 ccRCC cases and 72 cases of normal control for further analysis. In this current study, overall survival (OS) was the primary outcome. A total of 13 m6A-related genes contained writers (KIAA1429, METTL3, METTL14, RBM15, WTAP and ZC3H13), erasers (FTO and ALKBH5) and readers (YTHDF1, YTHDF2, HNRNPC, YTHDC1 and YTHDC2). By utilizing the R programming language, we did an overlap by comparing the standardized RNA-seq data with the m6A-related genes.

### Identification of differently expressed m6A-related genes

Differently expressed m6A-related genes between ccRCC and normal tissues were calculated by utilizing the R “Limma” package and adjusted P-value (FDR) less than 0.05 and at least twofold changes (FCs) were set as the threshold. Heatmap and boxplot were used to show the distribution and expression of differently expressed m6A-related genes respectively. Besides, the R “corrplot” package was used to calculate the pearson correlations between 13 m6A-related genes based on gene expression levels at the transcriptional level.

### Kaplan‐meier survival analysis and ROC curve of METTL14 in ccRCC

Based on the median expression of METTL14, ccRCC patients were divided into high-risk groups and low-risk groups. By means of Kaplan–Meier (K–M) method and the log-rank test, we evaluated the survival differences between these two groups. The receiver operating characteristic curves (ROC) analyses were performed by using the R “survivalROC” package and the area under the curve (AUC) values were calculated to assess the specificity and sensitivity of METTL14.

### Quantitative real‐time PCR (qRT-PCR)

The total RNA from ccRCC tissues and adjacent normal tissues was acquired using TRIzol Reagent (Invitrogen, Carlsbad, CA, USA) on the basis of a standard extraction protocol. The cDNA was synthesized by HiScript II (Vazyme, China), and qPCR analysis was performed on StepOne Plus Real-Time PCR system (Applied Biosystems, USA). The initial reaction was incubated at 95 °C for 10 min, followed by 40 cycles of 95 °C for 15 s and 60 °C for 1 min in accordance with the SYBR green method. The relative expression levels were analyzed by comparing 2^−ΔΔCT^ method. All reactions were carried out in triplicate. Actin was used for the internal reference. Primers were synthesized by TSINGKE (Beijing, China), including Actin (F:5’ ATGACTTAGTTGCGTTACACC 3’, R:5’ GACTTCCTGTAACAACGCATC 3’) and METTL14 (F:5’ TTTCTCTGGTGTGGTTCTGG 3’, R:5’ AAGTCTTAGTCTTCCCAGGATTG 3’). Six paired tumor tissues and their adjacent normal tissues were obtained from ccRCC patients from Affiliated Hospital of Nantong University. Ethical approval was obtained from the Institutional Research Ethics Committees of Affiliated Hospital of Nantong University and informed written consent was obtained from all of subjects.

### Univariate and multivariate cox hazard regression analyses

In order to identify independent prognostic factors, univariate and multivariate Cox regression analyses were utilized to exclude clinical characteristics with little prognostic values with OS from age, gender, race, grade, stage, staged T, staged N, staged M and METTL14 expression level by R package. Moreover, ROC curves of these nine factors and their AUC values were also calculated and compared by the R “survivalROC” package.

### The establishment of a nomogram for prognosis prediction

In order to predict the 1-, 3-, and 5-year survival probabilities, a nomogram was conducted to visualize the associations between survival rates of OS and individual predictors (age, gender, race, grade, stage, T, M, N and METTL14) by means of the R “rms” package. By using the point scale in the nomogram, every parameter was assigned a point and the whole points were calculated by summing up the points of all factors.

### Gene set enrichment analysis (GSEA)

GSEA is a systematic method used to figure out whether the hallmark gene sets predicted statistically significant differences between two different groups [22]. Here, GSEA was performed to analyze the significant survival differences between two groups divided by the expression of METTL14. The permutation tests were carried out 1000 times for discovering significant critical biological pathway. It had been considered to be significantly enriched that nominal p value less than 0.05 and FDR less than 25%.

### Prognostic model (riskscore, RS) construction and evaluation

By means of multivariate COX regression analysis, we excluded some clinical characteristics (gender, race, T, M, N) with little prognostic value. Moreover, based on the correlation coefficient of each significant clinical characteristic (age, grade, and stage) and METTL14, we established an independent prognostic index (RS) for predicting the OS of ccRCC patients. It was calculated using the following formula β_1_ × clinical characteristics_1_ + β_2_ × clinical characteristics_2_ + ····· + β_n_ × METTL14, where β was the correlation coefficient.

As for evaluation, ccRCC patients will be classified into low-risk and high-risk groups, according to the median risk score of our established prognostic model (RS). By means of Kaplan–Meier (K–M) method and the log-rank test, we evaluated the survival differences between these two groups. The ROC analyses were performed by using the R “survivalROC” package based on each prognostic model. We also displayed the risk score distribution of different risk groups and the number of censored patients.

### Statistical analysis

All statistical data and figures were analyzed by using SPSS 24.0 (IBM, Chicago, USA), R 3.3.1 (https://www.r-project.org/) and GraphPad Prism 6.0 (San Diego, CA, USA). The correlations between two different genes were analyzed by Pearson’s correlation method. The association between clinicopathologic parameters and METTL14 was estimated by the Wilcoxon signed rank test and logistic regression. The Kaplan-Meier curve and log-rank test were carried out to estimate survival predictive performance of METTL14 and RS. Univariate and multivariate Cox regression analyses were used to evaluate the relationship between variables and OS. A nomogram was also created using the rms package of R software. ROC and its AUC values were calculated to evaluate the prognostic ability of METTL14 or RS by using the package of “survivalROC” in R. All statistical results with p < 0.05 were considered statistically significant.

## Results

### Relative expression of m6A-related genes in ccRCC

**Fig. 1:**
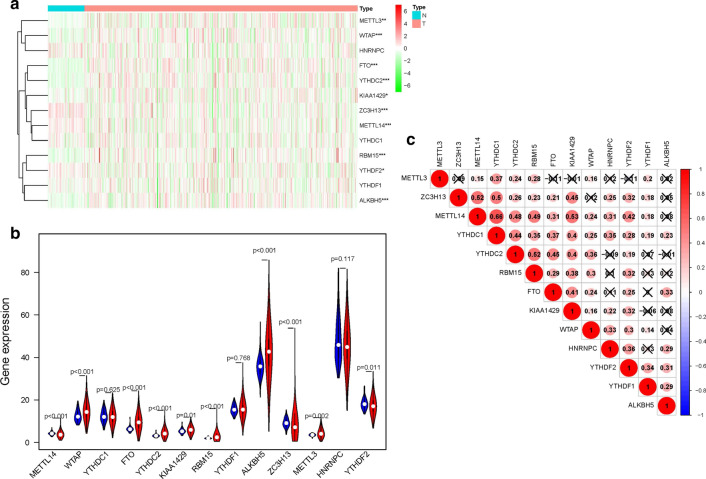
Relative expression of m6A-related genes in ccRCC; **a** Heatmap of ccRCC and normal tissues expressing the 13 m6A related genes in TCGA database; **b** boxplots of ccRCC and normal tissues expressing the 13 m6A related genes in TCGA database; **c** correlations of these 13 m6A related genes in the TCGA database

We analyzed the mRNA expression levels of 13 m6A-related genes (METTL3, METTL14, WTAP, RBM15, ZC3H13, KIAA1429, FTO, ALKBH5, YTHDC1, YTHDC2, YTHDF1, YTHDF2, HNRNPC) in TCGA database to identify the different expressed genes between ccRCC tissues and normal tissues. Figure 1a is the heat map which indicated the expression levels of m6A-related genes. The expression of each m6A-related gene in ccRCC tissues and adjacent normal tissues is displayed in Fig. 1b. We further investigated the correlation between each two types of m6A-related genes. As displayed in Fig. 1c, positive correlations were shown as red bubbles and negative correlations were shown as blue bubbles. The size of each bubble represented the P value. The result revealed that METTL14 and YTHDC1 were the two most relevant m6A-related genes (Pearson’s r = 0.66). METTL14 also had a positive correlation with KIAA1429 (Pearson’s r = 0.53) and ZC3H13 (Pearson’s r = 0.53), respectively.

### The expression of METTL14 in ccRCC and qRT-PCR verification

**Fig. 2:**
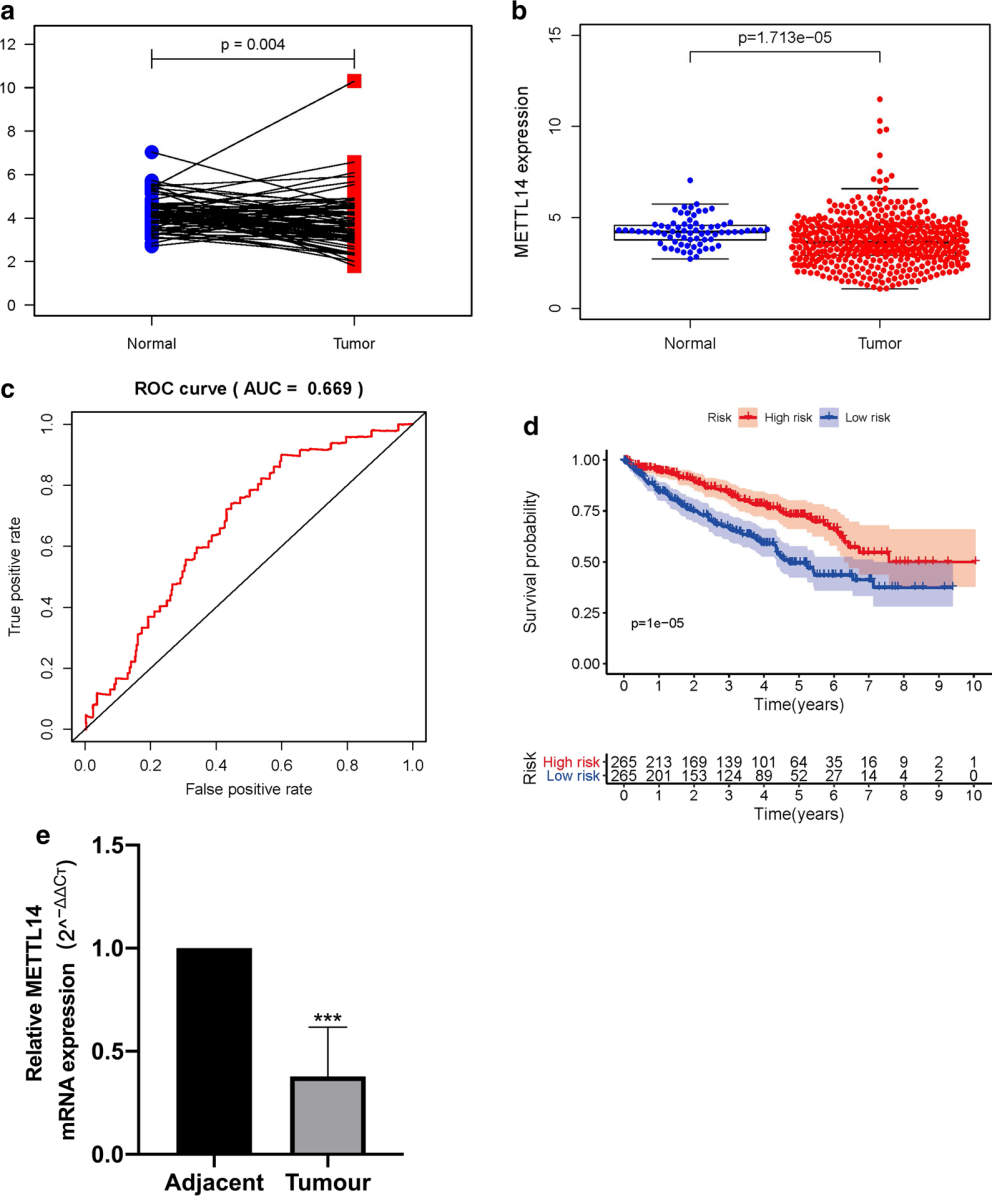
The expression of METTL14 in ccRCC tissues; **a** pairwise boxplot of the METTL14 expression between the ccRCC (n = 72) and matched normal tissues (n = 72) in TCGA dataset; **b** relative expression levels of the METTL14 expression between the ccRCC (n = 539) and normal tissues (n = 72) in TCGA dataset; **c** ROC curve performed to assess the OS predictive performance of METTL14 expression in TCGA dataset; **d** Kaplan-Meier curves for OS in the high-risk and low-risk groups when stratified by METTL14 expression; **e** qRT-PCR verification of METTL14 expression between ccRCC tissues (n = 6) and adjacent normal tissues (n = 6). The bar graphs represent means ± standard deviation. ***p < 0.001

As presented in 72 pairs of ccRCC and matched normal tissues, METTL14 had a low expression in tumor tissues in TCGA dataset (p = 0.004, Fig. 2a). Moreover, the mRNA expression level of METTL14 was significantly decreased in ccRCC tissues (n = 539) compared with that in normal tissues (n = 72) in Fig. 2b (p < 0.001). Receiver operating characteristic (ROC) curves for 5-year survival were carried out to demonstrate the performance of the METTL14 expression level for survival prediction (Fig. 2c), and its 5-year AUC was 0.669. The K-M curve indicated that patients in the low-METTL14 group had a shorter OS time than patients in the high-METTL14 group (p < 0.001, Fig. 2d). As showed in Fig. 2e and Additional file 1. Table S1, the qRT-PCR results verified that the expression of METTL14 was significantly down-regulated in the ccRCC tissues (n = 6) compared with adjacent normal tissues (n = 6; p < 0.001).

#### Association between METTL14 expression and clinicopathologic parameters

**Fig. 3:**
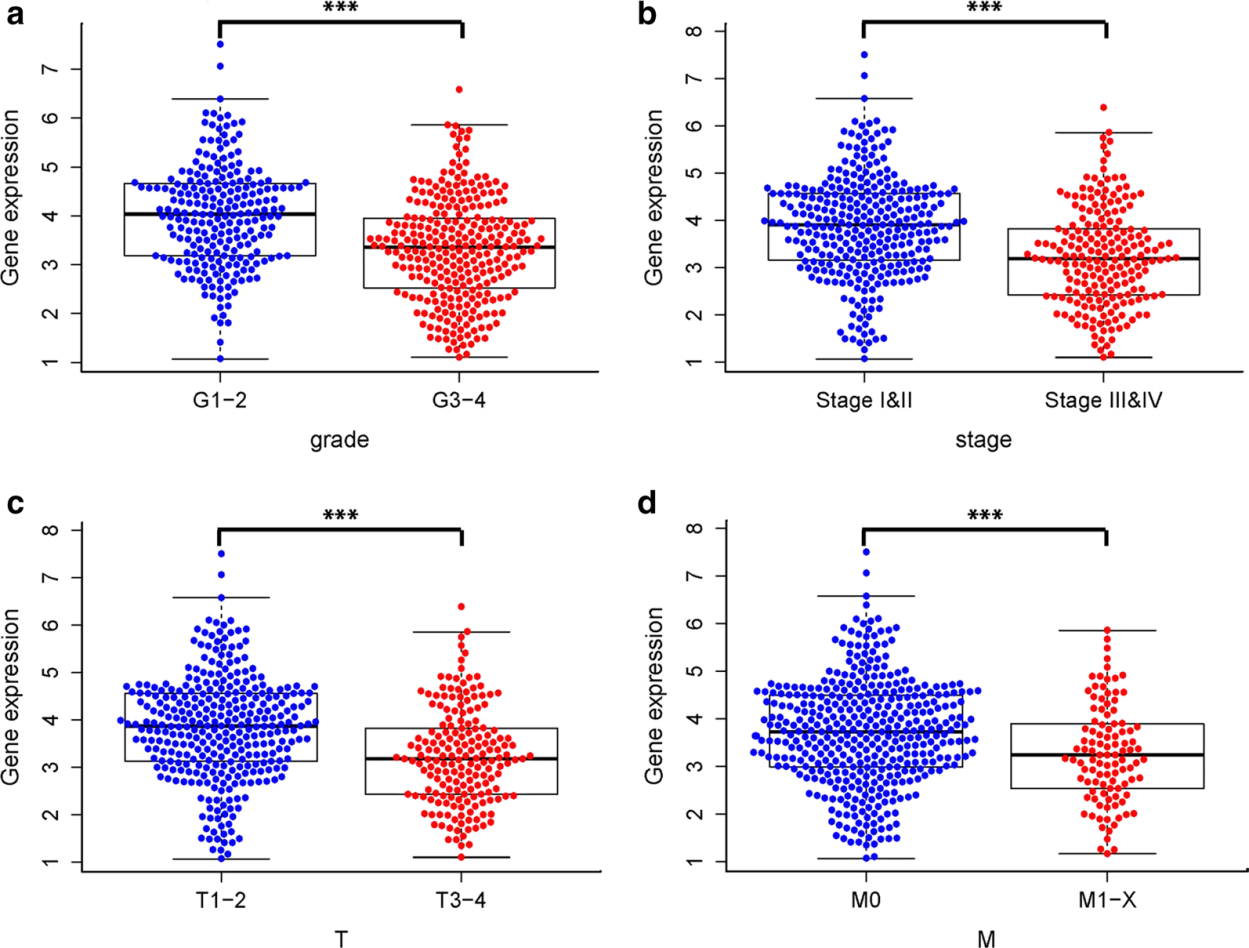
Associations between METTL14 expression and clinicopathologic characteristics; **a** Grade; **b** Stage; **c** T stages; **d** M stages. ***p < 0.001

The results of independent sample t-tests showed that the METTL14 expression was lower in high grade (p < 0.001; Fig. 3a), pathologic stage (p < 0.001; Fig. 3b), T stage (p < 0.001; Fig. 3c), and M stage (p < 0.001; Fig. 3d). In addition, there is no difference of METTL14 expression identified between male and female, Asian and Caucasian, or N0 stage and N I–III stage.

Based on logistic regression analysis, METTL14 expression was revealed to be associated with prognostic clinicopathologic characteristics (Table 1). High METTL14 expression in ccRCC patients is significantly (p < 0.05) associated with several clinicopathologic parameters including male (OR = 0.573), low grade (OR = 0.338), low stage (OR = 0.310), low T stage (OR = 0.318) and more distant metastasis (OR = 0.467). These analyses revealed that ccRCC patients with low METTL14 expression tend to progress to a more advanced grade and distant metastasis than those with high METTL14 expression.

**Table 1:** METTL14 expression associated with clinical pathological variables (logistic regression)

Variable	METTL14 mRNA expression		
	OR	95% CI	*p*
Age(year)			
> 65 vs. ≤ 65	0.769	0.532–1.110	0.161
Gender			
Female vs. male	0.573	0.395–0.827	*0.003*
Race			
African vs. White	0.923	0.198- 4.300	0.916
Asian vs. White	0.915	0.507–1.644	0.766
Grade			
G3–4 vs. G1–2	0.338	0.235–0.484	*0.000*
Stage			
Stage III & IV VS Stage I & II	0.310	0.213–0.449	*0.000*
T stage			
T3–4 vs. T1–2	0.318	0.216–0.464	*0.000*
N stage			
N1-X vs. NO	0.867	0.611–1.229	0.423
M stage			
M1-X vs. M0	0.467	0.297–0.725	*0.000*

### METTL14 could serve as an independent prognostic factor

**Fig. 4:**
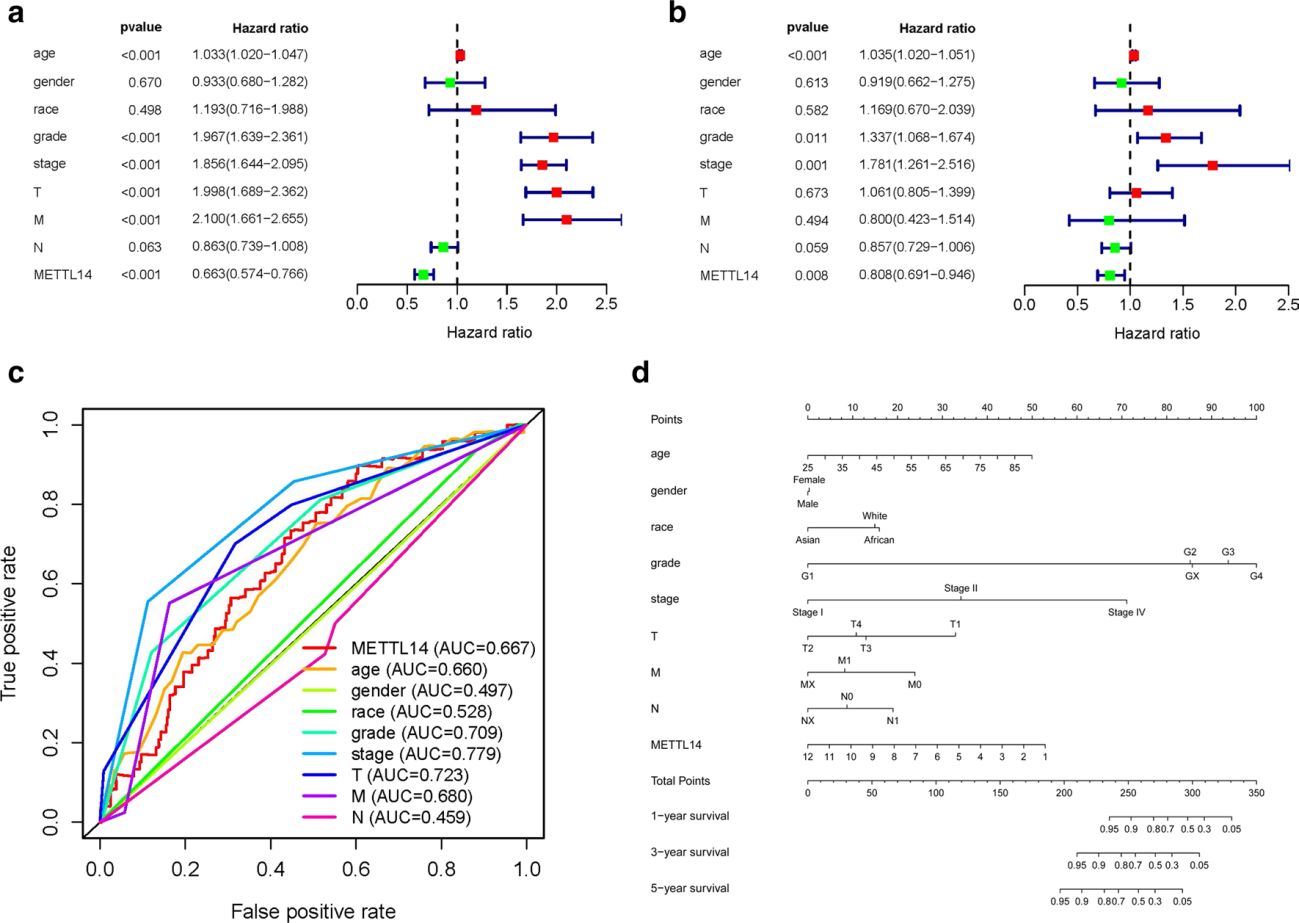
METTL14 could serve as an independent prognostic factor and established nomogram; **a**, **b** Univariate and multivariate cox regression analyses; **c** MultiROC analyses of OS for the METTL14 expression and classical clinicopathological parameters in TCGA dataset; **d** nomogram to predict the OS of ccRCC patients based on clinical parameters and METTL14 expression

Univariate Cox and multivariate Cox regression analyses were conducted to investigate whether the METTL14 expression was an independent factor correlated with OS (Table 2). Univariate Cox analysis revealed that the METTL14 expression, age, grade, pathological stage, T stage and M stage were all associated significantly with the overall survival of ccRCC patients (all p < 0.05; Fig. 4a). Multivariate Cox regression analysis showed high METTL14 expression correlated significantly with a better OS (HR = 0.808; p = 0.008). Other clinicopathological parameters correlated with prior overall survival consist of old age, low grade and advanced stage (Fig. 4b). Hence, our results indicated that the METTL14 expression might be an independent prognostic factor of OS when adjusted by those variables. Moreover, ROC curves for OS were also carried out to demonstrate the predictive ability of the METTL14 expression and clinicopathological variables (Fig. 4c). The AUC of the METTL14 expression (AUC = 0.667) was obviously higher than that of age (AUC = 0.660), gender (AUC = 0.497), race (AUC = 0.528) and lymph nodes status (AUC = 0.459). However, the AUC for METTL14 is lower than tumor grade (AUC = 0.709), pathological stage (AUC = 0.779), T stage (AUC = 0.779) and M stage (AUC = 0.680). These results demonstrated that it is insufficient to predict survival of clear cell renal cell carcinoma patients by using the expression level of METTL14 alone.

**Table 2:** Univariate and multivariate analyses of METTL14 mRNA level and clinicopathological variables predictive of overall survival

Variable	Univariate analysis			Multivariate analysis		
	HR	95% CI	*p*	HR	95% CI	*p*
Overall survival						
Age(year)	1.033	1.020–1.047	*0.000*	1.035	1.020–1.051	*0.000*
Gender	0.933	0.680–1.282	0.670	0.919	0.662–1.275	0.613
Race	1.193	0.716–1.988	0.498	1.169	0.670–2.039	0.582
Grade	1.967	1.6389-2.360	*0.000*	1.337	1.068–1.674	*0.011*
Stage	1.856	1.644–2.095	*0.000*	1.781	1.261–2.516	*0.001*
T	1.998	1.689–2.362	*0.000*	1.061	0.805–1.399	0.673
N	0.863	0.739–1.008	0.063	0.857	0.729–1.006	0.059
M	2.100	1.661–2.655	*0.000*	0.800	0.423–1.514	0.494
METTL14	0.663	0.574–0.766	*0.000*	0.808	0.691–0.946	*0.008*

Italicized values indicated that *p* < 0.05

*HR *hazard ratio, estimated from Cox proportional hazard regression model, *CI *confidence interval of the estimated HR; Multivariate models were adjusted for age, grade, race, stage and T,N,M classification

#### Establishment of ccRCC prognostic prediction nomogram

In order to predict the 1-, 3-, and 5-year survival probabilities, a nomogram was established based on eight clinicopathological parameters (age, gender, race, grade, stage, T, M, N) and METTL14 gene (Fig. 4d). By using the point scale in the nomogram, every parameter was assigned a point and the whole points were calculated by summing up the points of all factors. Besides, we could calculate survival rates of ccRCC patients at 1-, 3-, and 5-year, which might improve the predictive method by making it more intuitive.

#### GSEA identified METTL14 -related signaling pathways

**Fig. 5:**
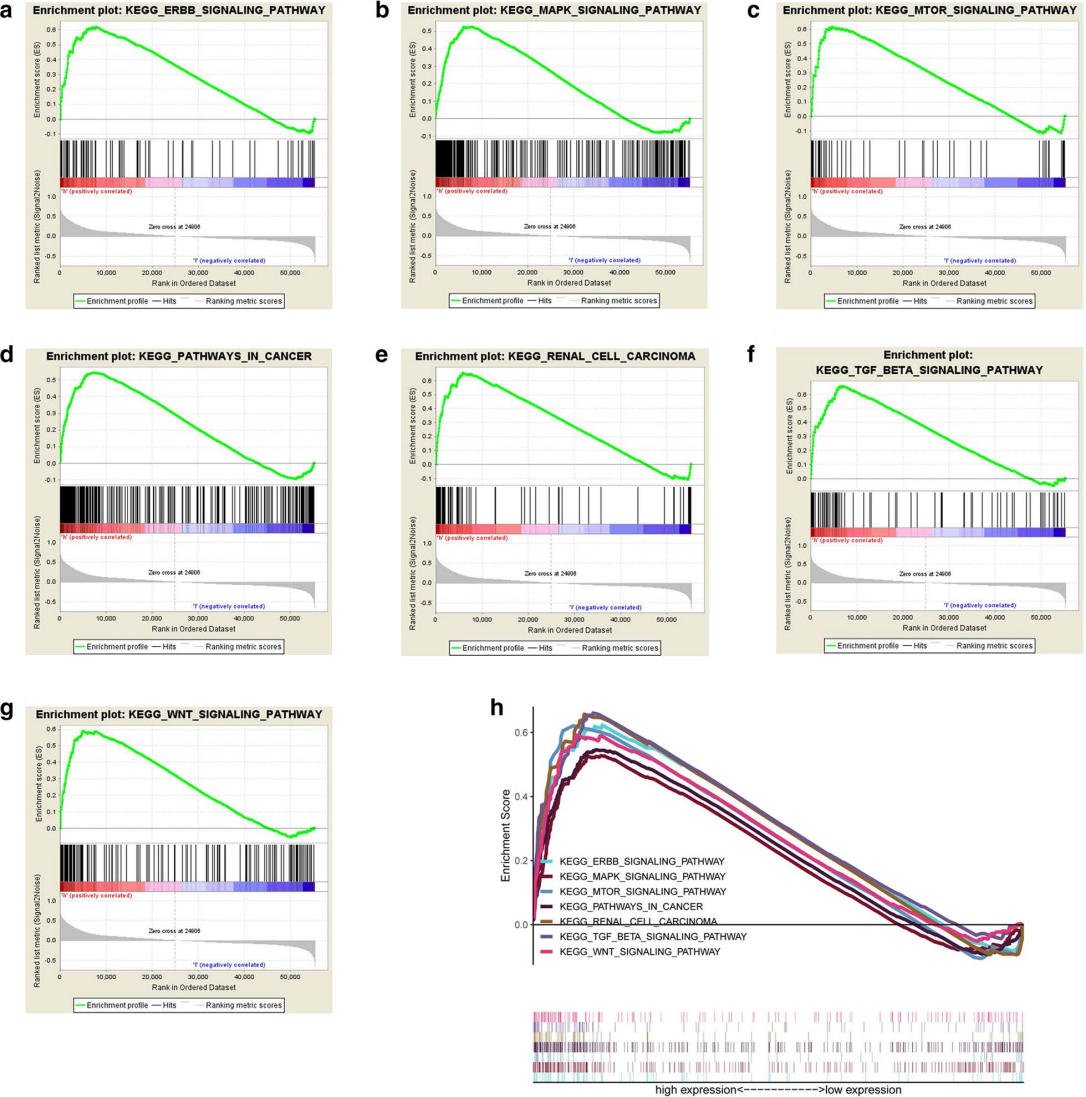
Enrichment plots from gene set enrichment analysis (GSEA); **a** ERBB pathway; **b** MAPK pathway; **c** MTOR pathway; **d** pathway in cancer; **e** renal cell carcinoma; **f** TGF-β pathway; **g** Wnt pathway; **h** The seven most significantly enriched signaling pathways

In consideration of METTL14 as an independent prognostic factor of OS for ccRCC patients, we were eager to explore how METTL14 was involved in ccRCC pathogenesis. We carried out Gene Set Enrichment Analysis (GSEA) between tissues with different METTL14 expression levels. Based on the normalized enrichment score (NES) and FDR q-val (FDR < 0.01), we selected the most significantly enriched biological pathways. The results indicated that high METTL3 expression was associated with some essential signaling pathways including ERBB pathway, MAPK pathway, mTOR pathway, renal cell carcinoma, pathway in cancer, TGF-β pathway and Wnt pathway (Fig. 5 and Table 3), giving a clue of the underlying mechanism in the pathogenesis of ccRCC.

**Table 3:** Gene sets enrichment analysis of high METTL14 mRNA expression level in the ccRCC cohort

Gene set name	NES	NOM p-val	FDR q-val
KEGG_ERBB_SIGNALING_PATHWAY	2.346	0.000	0.001
KEGG_MAPK_SIGNALING_PATHWAY	2.384	0.000	0.001
KEGG_MTOR_SIGNALING_PATHWAY	2.295	0.000	0.001
KEGG_PATHWAYS_IN_CANCER	2.279	0.000	0.001
KEGG_RENAL_CELL_CARCINOMA	2.279	0.000	0.001
KEGG_TGF_BETA_SIGNALING_PATHWAY	2.284	0.000	0.001
KEGG_WNT_SIGNALING_PATHWAY	2.312	0.000	0.001

*NES *normalized enrichment score, *NOM *nominal, *FDR *false discovery rate. Gene sets with NOM p-val < 0.01 and FDR q-val < 0.01 are considered as significant

#### Construction and evaluation of the RS

Based on the multivariate Cox regression analysis with TCGA dataset, METTL14 expression and three clinicopathological variables consisting of age, pathologic stage, and histological grade were indicated as independent prognostic parameters (p < 0.05) (Table 2). Thus, METTL14 expression and three clinicopathological variables including age, pathologic stage, and histological grade were integrated to develop the riskscore (RS) that could predict the prognosis of ccRCC by using a multivariate Cox regression model (Table 4). The formula of RS was as follows: RS=(0.036132 × age) + (0.310219 × grade) + (0.509547 × stage) + (− 0.20258 × METTL14 expression).The ccRCC patients in TCGA dataset were divided into high-risk and low-risk groups by the median expression value of RS.

**Table 4:** Establishment of a prognostic index (riskscore, RS) based on METTL14 expression and clinical characteristics by multivariate cox regression analysis for predicting ccRCC patients’ overall survival

Id	Coef	HR	HR.95L	HR.95H	p value
Age	0.036132	1.036792	1.021773	1.052033	1.22E−06
Grade	0.310219	1.363723	1.091211	1.704291	0.006384
Stage	0.509547	1.664537	1.450941	1.909576	3.54E−13
METTL14	-0.20258	0.816622	0.699427	0.953454	0.010377

*HR *hazard ratio, estimated from Cox proportional hazard regression model, *CI *confidence interval of the estimated HR

**Fig. 6:**
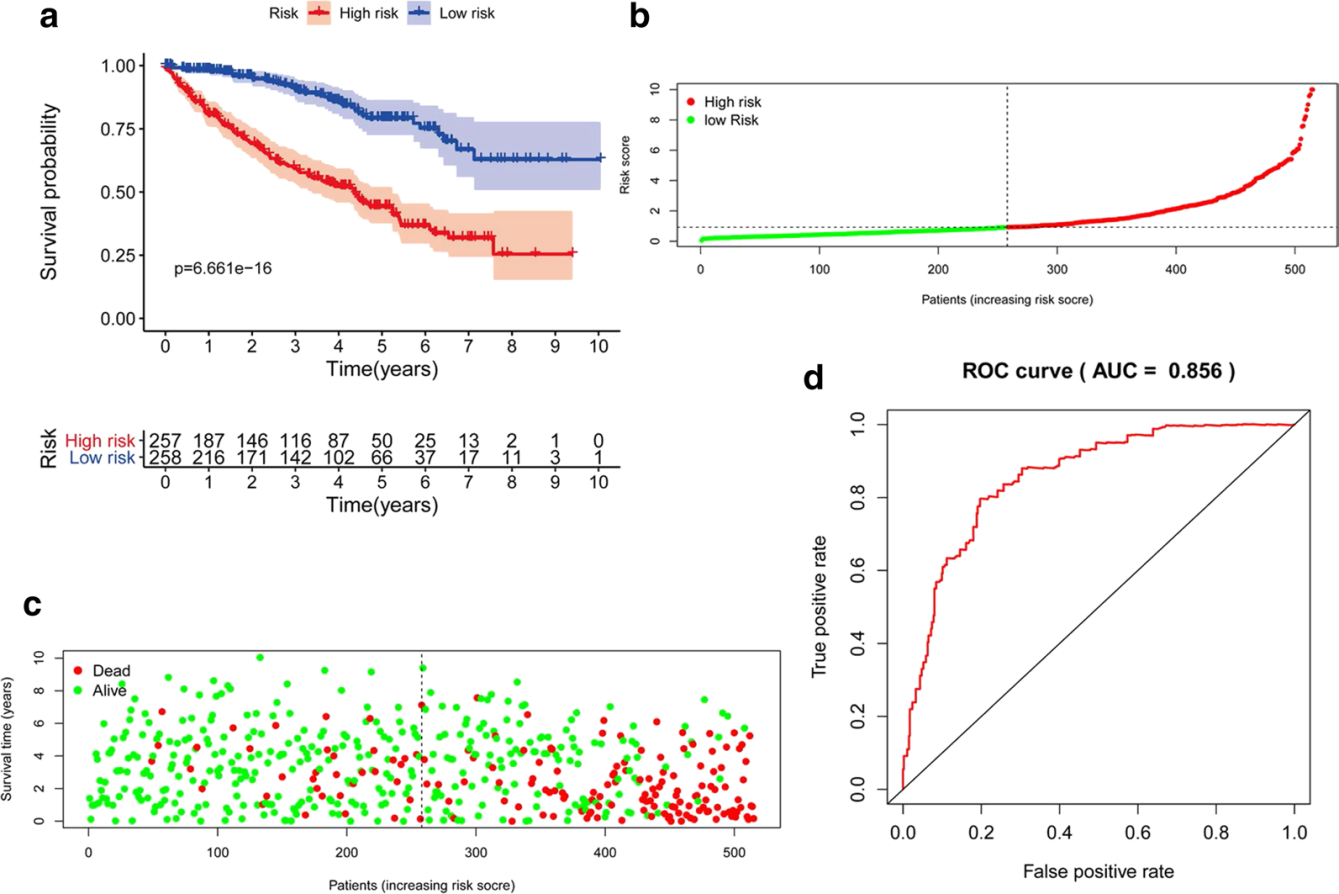
Evaluation of the prognostic index (riskscore, RS) based on METTL14 expression and clinical characteristics in TCGA dataset; **a** Kaplan–Meier plot represents that patients in the high-risk group had significantly shorter overall survival time than those in the low-risk group; **b** the risk score distribution of patients in the TCGA dataset; **c** the higher the risk scores, the higher the numbers of dead persons; **d** ROC curve analysis for survival prediction by the RS based on the TCGA database

In order to verify the performance of RS in predicting the OS of ccRCC, we constructed a Kaplan-Meier curve to analyze the different survival time between the high- and low-risk groups. Patients in the low-risk group had much better OS compared with that in the high-risk group (P < 0.001, Fig. 6a). Figure 6b, c indicated that the higher the risk scores, the higher the patients in high-risk subgroups, and the higher the numbers of dead persons. In order to validate the prognostic predictive performance of RS for ccRCC, we also performed a ROC curve analysis based on data in the TCGA dataset. The AUC for the RS was 0.856 (Fig. 6d), indicating the satisfactory performance of the RS for prognostic prediction.

#### Correlations between METTL14 and related genes

**Fig. 7:**
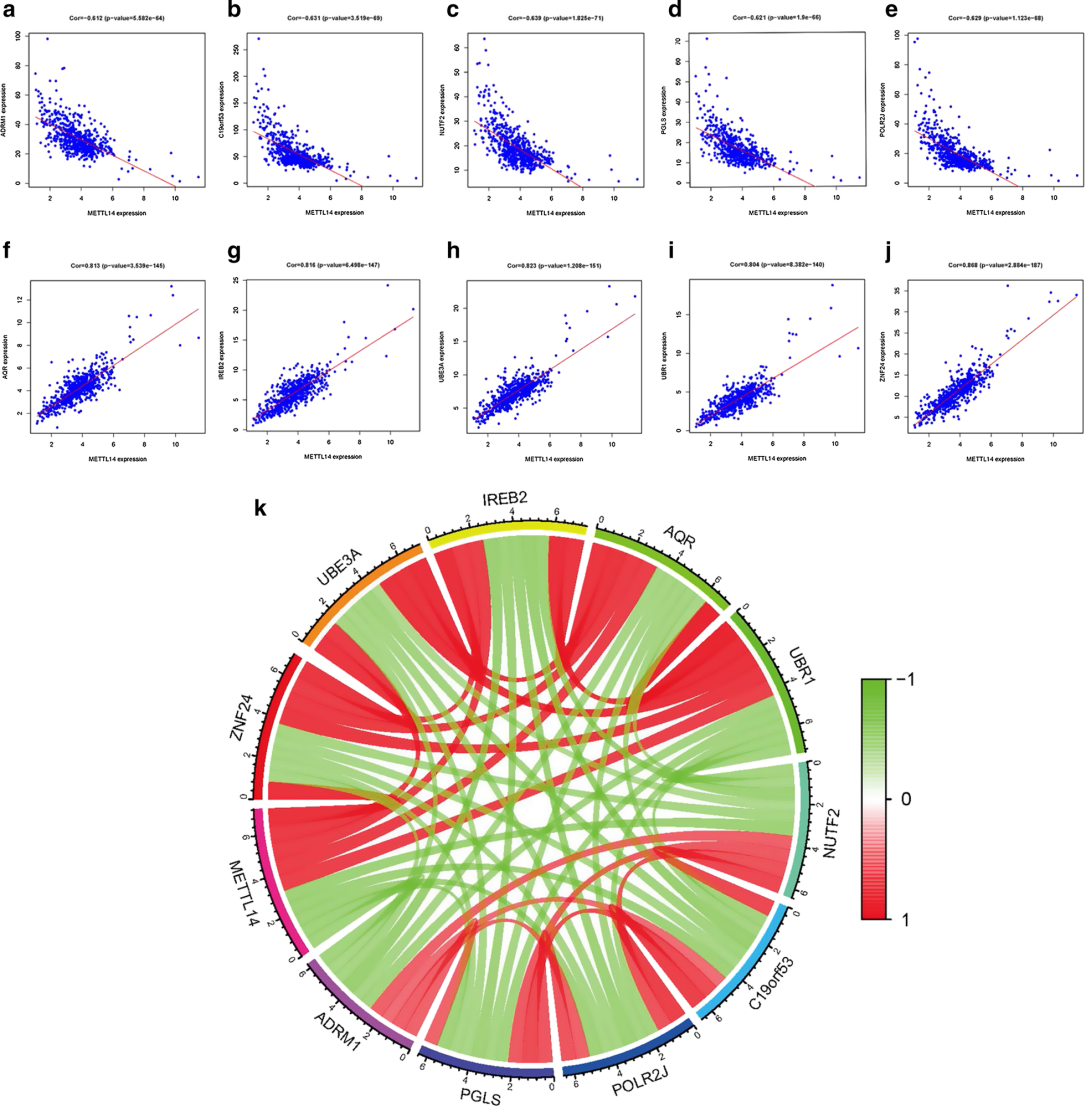
Correlations between METTL14 and related genes; The expression of METTL14 in ccRCC samples had negative correlations with **a** ADRM1; **b** C19orf53; **c** NUTF2; **d** PGLS; **e** POLR2J; The expression of METTL14 in ccRCC samples had positive correlations with **f** AQR; **g** IRE82; **h** UBE3A; **i** UBR1; **j** ZNF24; **k** Correlations of METTL14 and 10 METTL14-related genes in TCGA dataset

Figure 7 indicated the relationship of METTL14 and related genes based on TCGA dataset. As shown in Fig. 7a−e, the five most negatively relevant genes are NUTF2 (Pearson’s r = − 0.639, p < 0.001), C19orf53 (Pearson’s r = − 0.631, p < 0.001), POLR2J (Pearson’s r = − 0.629, p < 0.001), PGLS (Pearson’s r = − 0.621, p < 0.001), and ADRM1 (Pearson’s r = − 0.612, p < 0.001). In contrast, the five most negatively relevant genes are ZNF24 (Pearson’s r = 0.868, p < 0.001), UBE3A (Pearson’s r = 0.823, p < 0.001), IRE82 (Pearson’s r = 0.816, p < 0.001), AQR (Pearson’s r = 0.813, p < 0.001), and UBR1 (Pearson’s r = 0.804, p < 0.001) (Fig. 7f−j). Besides, Fig. 7k also indicated the associations between METTL14 and five most negatively relevant genes, as well as five most negatively relevant genes. Five most negatively relevant genes (ZNF24, UBE3A, UBE3A, IRE82, AQR and UBR1) were positively associated with each other and negatively related with five most negatively relevant genes (NUTF2, C19orf53, POLR2J, PGLS, ADRM1).

## Discussion

Recently, more and more research has focused on the field of cancer [23]. As the most common histological type of RCC, clear cell renal cell carcinoma accounts for 90% of kidney neoplasms [24]. Although the development of operational methods and medical oncology, it remained eager to explore new reliable prognostic targets for survival prediction and treatment of ccRCC. Recently, some studies have reported the pivotal roles that m6A methylation played in tumorigenesis and development [4, 25]. However, few researches focused on the association between m6A methylation regulatory genes and the prognostic prediction in ccRCC. Hence, our present study focused on the prognostic role of METTL14 in ccRCC.

In the current study, we explored the association among METTL14 expression, clinicopathologic parameters and patient survival based on TCGA database. The results revealed that METTL14 showed obviously lower expression in ccRCC tissues than that in adjacent normal tissues. Additionally, high METTL14 expression was significantly associated with better pathologic stage, histological grade, T stage, M stage, and satisfactory survival time. The METTL14 expression level was proved to be an independent predictive factor of overall survival for ccRCC patients. In order to explore how METTL14 was involved in ccRCC pathogenesis, we carried out GSEA between tissues with different METTL14 expression levels and found that high METTL14 expression was associated with some essential signaling pathways including ERBB pathway, MAPK pathway, mTOR pathway, renal cell carcinoma, pathway in cancer, TGF-β pathway and Wnt pathway. We further constructed a risk score that could predict the prognosis of ccRCC by combining the METTL14 expression with three clinicopathological variables including age, pathologic stage, and histological grade. Then, we confirmed the performance of RS in the TCGA database and the AUC for the RS was 0.856, which indicated the satisfactory ability of the RS for prognostic prediction. Finally, we investigated the relationship of METTL14 and related genes based on TCGA dataset and discovered that the five most negatively relevant genes are NUTF2, C19orf53, POLR2J, PGLS, and ADRM1. In contrast, the five most negatively relevant genes are ZNF24, UBE3A, IRE82, AQR, and UBR1.

Given the results of correlation analysis based on TCGA cohort, METTL14 and YTHDC1 were the two most relevant m6A regulatory genes (Pearson’s r = 0.66), which indicated that the process of RNA m6A modification in ccRCC may be the result of combined action by m6A writer gene (METTL14) and reader gene (YTHDC1). Recently, a study suggested that numerous concurrence of CNVs in two m6a regulatory genes was observed in ccRCC, hence, m6A writer gene and reader gene may have synergistic effects on tumorigenesis and progression [25]. Unlike METTL3, METTL14 have been demonstrated to show tumor-suppressive functions in most types of cancer. In hepatocellular carcinoma, reduced METTL14 expression, but not METTL3 was associated with adverse patient prognosis and tumor metastasis by regulating the process of pri-miR126 [20]. A similar finding is indicated for glioblastoma stem cells, METTL14 knockdown could enhance glioblastoma stem cell growth, self-renewal and tumorigenesis by enhancing m6A modification of ADAM19 and decreasing its expression, suggesting a tumor suppressor role of METTL14 [26]. Recently, it is found that mutation of METTL14 in endometrial cancer cells could down regulate m6A mRNA methylation and enhance proliferation and tumorigenicity by regulating AKT activity [27]. In contrast, one recent study demonstrated that high METTL14 expression is present in normal hematopoietic stem/progenitor cells (HSPCs) and acute myelogenous leukemia (AML) cells with t(11q23), t(15;17) or t(8;21) translocation, and suppresses terminal myeloid differentiation and promotes leukemogenesis by positively regulating expression of MYB and MYC through m6A-based post-transcriptional regulation [28].

In our study, the results demonstrated that low METTL14 expression was significantly associated with poor clinical pathological variables and adverse survival time. Hence, METTL14 expression may be a potential prognostic marker of favorable survival and a tumor suppressor in ccRCC. Similar as our results, a previous study revealed that METTL14 could suppress P2RX6 mRNA and protein level. Moreover, the m6A-suppressed P2RX6 activation could promote renal cancer cells migration and invasion through ATP-induced Ca2 + influx modulating ERK1/2 phosphorylation and MMP9 signaling pathway [29].

Our results discovered that high METTL14 expression had association with ERBB pathway, MAPK pathway, mTOR pathway, renal cell carcinoma, TGF-β pathway and Wnt pathway by GSEA. These pathways are crucial biological processes in tumorigenesis and development of ccRCC. As we all know, epidermal growth factor receptor (EGFR), as known as ErbB1, plays vital roles in enhancing tumor cell proliferation, suppressing apoptosis and contributing to the development and metastasis of renal cell carcinoma [30–32], and translation of EGFR has been recently found to be promoted by METTL3 in cancer cells [19]. A previous study reported an integrated molecular study of ccRCC (106 ccRCC cases). mTOR was found to be mutated in 6 cases [33]. Furthermore, 26 percent of ccRCC cases were discovered to have mutations involving in PI3K/AKT/mTOR signaling. Nishida et al. [34] revealed that loss of TGFBR3, one of the TGF-β pathway molecular, could enhance metastatic abilities in ccRCC through TGF-β-dependent pathway. However, the relationship between METTL14 expression with these signaling pathways is the first to be reported, and the regulatory mechanism needs to be further investigated. In addition, Wnt pathway is another important pathway associated with high METTL14 expression in ccRCC by GSEA. A recent study proved knockdown of m6A writer gene METTL14 in gastric cancer cell enhanced proliferation and invasiveness by Wnt and PI3K-Akt signaling [35]. Therefore, further investigations need to be carried out to explore that whether progression in ccRCC could be affected by METTL14 through Wnt/PI3K-Akt signaling pathway.

## Conclusions

Taken together, Our results indicated that METTL14 could serve as a favorable prognostic factor for ccRCC. Moreover, ERBB pathway, MAPK pathway, mTOR pathway, TGF-β pathway and Wnt pathway might be the primary pathways regulated by METTL14. Furthermore, we also developed a risk score (RS) to predict the OS of ccRCC patients by combining METTL14 expression and clinicopathological variables and confirmed the performance of the RS model in TCGA dataset. Further prospective experiments should be carried out to explore molecular mechanisms of METTL14 and estimate the clinical application of our established RS model in ccRCC (Additional file 1).

## Supplementary information

**Additional file 1.** **Table S1:** Original file for qRT-PCR results.

## Data Availability

The RNA-sequencing data and corresponding clinical information were downloaded from The Cancer Genome Atlas (TCGA) database (https://portal.gdc.cancer.gov/).

## References

[1] Siegel RL, Miller KD, Jemal A (2020). Cancer statistics, 2020. Cancer J Clin.

[2] Yu W, Wang Y, Jiang Y, Zhang W, Li Y (2017). Genetic analysis and clinicopathological features of ALK-rearranged renal cell carcinoma in a large series of resected Chinese renal cell carcinoma patients and literature review. Histopathology.

[3] Wu J, Zhang P, Zhang G, Wang H, Gu W, Dai B, Zhang H, Shi G, Shen Y, Zhu Y (2017). Renal cell carcinoma histological subtype distribution differs by age, gender, and tumor size in coastal Chinese patients. Oncotarget.

[4] Chen T, Hao YJ, Zhang Y, Li MM, Wang M, Han W, Wu Y, Lv Y, Hao J, Wang L (2015). m(6)A RNA methylation is regulated by microRNAs and promotes reprogramming to pluripotency. Cell Stem Cell.

[5] Globisch D, Pearson D, Hienzsch A, Bruckl T, Wagner M, Thoma I, Thumbs P, Reiter V, Kneuttinger AC, Muller M (2011). Systems-based analysis of modified tRNA bases. Angew Chem.

[6] Dominissini D, Moshitch-Moshkovitz S, Schwartz S, Salmon-Divon M, Ungar L, Osenberg S, Cesarkas K, Jacob-Hirsch J, Amariglio N, Kupiec M (2012). Topology of the human and mouse m6A RNA methylomes revealed by m6A-sEq. Nature.

[7] Meyer KD, Saletore Y, Zumbo P, Elemento O, Mason CE, Jaffrey SR (2012). Comprehensive analysis of mRNA methylation reveals enrichment in 3’ UTRs and near stop codons. Cell.

[8] Meyer KD, Jaffrey SR (2014). The dynamic epitranscriptome: N6-methyladenosine and gene expression control. Nat Rev Mol Cell Biol.

[9] Liu N, Dai Q, Zheng G, He C, Parisien M, Pan T (2015). N(6)-methyladenosine-dependent RNA structural switches regulate RNA-protein interactions. Nature.

[10] Fu Y, Dominissini D, Rechavi G, He C (2014). Gene expression regulation mediated through reversible m(6)A RNA methylation. Nat Rev Genet.

[11] Lee M, Kim B, Kim VN (2014). Emerging roles of RNA modification: m(6)A and U-tail. Cell.

[12] Geula S, Moshitch-Moshkovitz S, Dominissini D, Mansour AA, Kol N, Salmon-Divon M, Hershkovitz V, Peer E, Mor N, Manor YS (2015). Stem cells. m6A mRNA methylation facilitates resolution of naive pluripotency toward differentiation. Science.

[13] Vu LP, Pickering BF, Cheng Y, Zaccara S, Nguyen D, Minuesa G, Chou T, Chow A, Saletore Y, MacKay M (2017). The N(6)-methyladenosine (m(6)A)-forming enzyme METTL3 controls myeloid differentiation of normal hematopoietic and leukemia cells. Nat Med.

[14] Lin Z, Hsu PJ, Xing X, Fang J, Lu Z, Zou Q, Zhang KJ, Zhang X, Zhou Y, Zhang T (2017). Mettl3-/Mettl14-mediated mRNA N(6)-methyladenosine modulates murine spermatogenesis. Cell Res.

[15] Fischer J, Koch L, Emmerling C, Vierkotten J, Peters T, Bruning JC, Ruther U (2009). Inactivation of the Fto gene protects from obesity. Nature.

[16] Haussmann IU, Bodi Z, Sanchez-Moran E, Mongan NP, Archer N, Fray RG, Soller M (2016). m(6)A potentiates Sxl alternative pre-mRNA splicing for robust Drosophila sex determination. Nature.

[17] Yu J, Chen M, Huang H, Zhu J, Song H, Zhu J, Park J, Ji SJ (2018). Dynamic m6A modification regulates local translation of mRNA in axons. Nucleic Acids Res.

[18] Zhang C, Zhi WI, Lu H, Samanta D, Chen I, Gabrielson E, Semenza GL (2016). Hypoxia-inducible factors regulate pluripotency factor expression by ZNF217- and ALKBH5-mediated modulation of RNA methylation in breast cancer cells. Oncotarget.

[19] Lin S, Choe J, Du P, Triboulet R, Gregory RI (2016). The m(6)A methyltransferase METTL3 promotes translation in human cancer cells. Mol Cell.

[20] Ma JZ, Yang F, Zhou CC, Liu F, Yuan JH, Wang F, Wang TT, Xu QG, Zhou WP, Sun SH (2017). METTL14 suppresses the metastatic potential of hepatocellular carcinoma by modulating N(6) -methyladenosine-dependent primary MicroRNA processing. Hepatology.

[21] Zhang Y, He W, Zhang S (2019). Seeking for correlative genes and signaling pathways with bone metastasis from breast cancer by integrated analysis. Front Oncol.

[22] Subramanian A, Tamayo P, Mootha VK, Mukherjee S, Ebert BL, Gillette MA, Paulovich A, Pomeroy SL, Golub TR, Lander ES (2005). Gene set enrichment analysis: a knowledge-based approach for interpreting genome-wide expression profiles. Proc Natl Acad Sci U S A.

[23] Sen Z, Zhan XK, Jing J, Yi Z, Wanqi Z (2013). Chemosensitizing activities of cyclotides from Clitoria ternatea in paclitaxel-resistant lung cancer cells. Oncol Lett.

[24] Cohen HT, McGovern FJ (2005). Renal-cell carcinoma. N Engl J Med.

[25] Chen M, Wei L, Law CT, Tsang FH, Shen J, Cheng CL, Tsang LH, Ho DW, Chiu DK, Lee JM (2018). RNA N6-methyladenosine methyltransferase-like 3 promotes liver cancer progression through YTHDF2-dependent posttranscriptional silencing of SOCS2. Hepatology.

[26] Cui Q, Shi H, Ye P, Li L, Qu Q, Sun G, Sun G, Lu Z, Huang Y, Yang CG (2017). m(6)A RNA methylation regulates the self-renewal and tumorigenesis of glioblastoma stem cells. Cell Rep.

[27] Liu J, Eckert MA, Harada BT, Liu SM, Lu Z, Yu K, Tienda SM, Chryplewicz A, Zhu AC, Yang Y (2018). m(6)A mRNA methylation regulates AKT activity to promote the proliferation and tumorigenicity of endometrial cancer. Nat Cell Biol.

[28] Weng H, Huang H, Wu H, Qin X, Zhao BS, Dong L, Shi H, Skibbe J, Shen C, Hu C (2018). METTL14 inhibits hematopoietic stem/progenitor differentiation and promotes leukemogenesis via mRNA m(6)A modification. Cell Stem Cell.

[29] Gong D, Zhang J, Chen Y, Xu Y, Ma J, Hu G, Huang Y, Zheng J, Zhai W, Xue W (2019). The m(6)A-suppressed P2RX6 activation promotes renal cancer cells migration and invasion through ATP-induced Ca(2+) influx modulating ERK1/2 phosphorylation and MMP9 signaling pathway. J Exp Clin Cancer Res CR.

[30] Staehler M, Rohrmann K, Haseke N, Stief CG, Siebels M (2005). Targeted agents for the treatment of advanced renal cell carcinoma. Curr Drug Targets.

[31] Han W, Lo HW (2012). Landscape of EGFR signaling network in human cancers: biology and therapeutic response in relation to receptor subcellular locations. Cancer Lett.

[32] Wee P, Wang Z (2017). Epidermal growth factor receptor cell proliferation signaling pathways. Cancers.

[33] Sato Y, Yoshizato T, Shiraishi Y, Maekawa S, Okuno Y, Kamura T, Shimamura T, Sato-Otsubo A, Nagae G, Suzuki H (2013). Integrated molecular analysis of clear-cell renal cell carcinoma. Nat Genet.

[34] Nishida J, Miyazono K, Ehata S (2018). Decreased TGFBR3/betaglycan expression enhances the metastatic abilities of renal cell carcinoma cells through TGF-beta-dependent and -independent mechanisms. Oncogene.

[35] Zhang C, Zhang M, Ge S, Huang W, Lin X, Gao J, Gong J, Shen L (2019). Reduced m6A modification predicts malignant phenotypes and augmented Wnt/PI3K-Akt signaling in gastric cancer. Cancer Med.

